# NIA START-UP CHALLENGE AND ACCELERATOR: EMPOWERING AGING RESEARCHERS FOR ENTREPRENEURIAL SUCCESS

**DOI:** 10.1093/geroni/igae098.0488

**Published:** 2024-12-31

**Authors:** Joy Toliver

**Affiliations:** National Institute on Aging, Baltimore, Maryland, United States

## Abstract

As an effort to support the development of aging researchers transitioning into entrepreneurship, the National Institute on Aging designed and launched its Start-Up Challenge and Accelerator. Through a five-month accelerator program, participants learn how to translate their research-driven solutions into market-ready products that promote healthy aging. Participants receive personalized mentorship, entrepreneurial training, networking opportunities, and a chance to win NIA funding. With cohorts in 2022 and 2023, the Startup Challenge and Accelerator supported the development of 40 transitioning entrepreneurs that are developing innovations to enhance the health and/or care of older adults and awarded cash prizes to eleven. Since the completion of the first cohort, graduates of the program have hit a multitude of milestones including, but not limited to, a company exit, raising venture capital financing, launching health system pilots, securing key strategic partners, and winning national pitch competitions. By attending this panel, you will learn more about the program, how to apply, and how past participants have leveraged the program to fuel their entrepreneurial growth and establish a path to success.

